# Atomic-Scale Fabrication of Micro/Nano Fe-Cu Galvanic Couples for Efficient Phenol Degradation

**DOI:** 10.3390/ma18235362

**Published:** 2025-11-28

**Authors:** Xiang Zhang, Xiudong Yu, Zhaoyang Li, Haishun Liu, Xiang Xiong, Changjiu Chen, Weiming Yang

**Affiliations:** 1 School of Materials Science and Physics, China University of Mining and Technology, Xuzhou 221116, China; x.zhang@cumt.edu.cn (X.Z.); xiudong_yu@tianma.cn (X.Y.); 15505165730@163.com (Z.L.); cjchen@cumt.edu.cn (C.C.); 2 Xingyuan Science and Technology Innovation Center, Guixi 335400, China; 3 State Key Laboratory of Powder Metallurgy, Central South University, Changsha 410083, China; 4 School of Mechanics and Civil Engineering, China University of Mining and Technology, Xuzhou 221116, China; wmyang@cumt.edu.cn

**Keywords:** atomic-scale fabrication, Fe-based amorphous alloys, advanced oxidation process, Fenton-like reactions, phenol, galvanic couples

## Abstract

Phenol, an essential feedstock widely used in manufacturing and chemical industries, inevitably results in the discharge of phenol-laden wastewater. To enhance the phenol-degradation efficiency of Fe-based amorphous alloys, a novel atomic-scale fabrication approach for Fe-Cu galvanic couples is proposed, enabling the rapid and uniform formation of micro/nano Fe-Cu structures on the surface of Fe-based alloys with significant improvement in the catalytic activity towards phenol. Micron/nano Fe-Cu couples can be fabricated within 15 s at 45 °C. Phenol degradation experiments reveal that the pristine amorphous alloy exhibits a 40 min hatching period before the phenol removal process, and it exhibits a kinetic constant (*k_obs_*) of 0.1596 min^−1^ after the hatching period, under conditions of 50 °C, 0.5 g/L catalytic loading, 10 mmol/L H_2_O_2_, and pH = 3 towards a 50 mg/L phenol solution. With the micro/nano Fe-Cu galvanic couples, the *k_obs_* value markedly increased to 2.23~2.36 min^−1^ under identical conditions except for 3 mmol/L H_2_O_2_, corresponding to approximately a 14-fold improvement. This cost-effective and time-efficient atomic-scale fabrication strategy offers a promising platform for the development of next-generation catalytic alloys and functional materials.

## 1. Introduction

Phenol (C_6_H_5_OH) is a highly toxic and widely distributed aromatic compound commonly detected in the effluents of petrochemical, pharmaceutical, pesticide, and coal chemical industries [1,2]. Its molecular structure, composed of a benzene ring and a hydroxyl group, imparts strong chemical stability and low biodegradability [3]. Consequently, phenol is recognized as a typical persistent organic pollutant (POP) [4]. Once discharged into natural water bodies, it can rapidly disperse through hydrological systems, leading to long-term contamination and severe ecological disruption [5,6].

With the growing scarcity and degradation of freshwater resources, phenolic pollution has become a critical environmental challenge, especially in regions with limited water availability [7]. The rapid expansion of the coal chemical industry has further intensified this issue. Coal chemical wastewater is characterized by a high concentration of toxic and refractory organic compounds, among which phenolic species are particularly problematic [8]. Even trace amounts of phenol can render water unsuitable for drinking or industrial purposes. Phenol exhibits both acute and chronic toxicity toward aquatic organisms—acute toxicity causes immediate lethality, while chronic exposure results in bioaccumulation and ecological imbalance [9]. In addition, its volatility enables it to transfer into the atmosphere or accumulate through the food chain, ultimately posing severe risks to human health [10]. Long-term exposure to phenol can damage the liver, kidneys, and nervous system, leading to cognitive impairment and even carcinogenesis [11]. Accordingly, phenol has been classified as a highly toxic substance by environmental authorities in China, the United States, and the European Union [12].

Despite its hazardous nature, phenol remains an indispensable industrial raw material in the manufacture of resins, plastics, and pharmaceuticals [13]. Consequently, phenol-containing wastewater is unavoidable, and its efficient treatment remains a major environmental challenge. According to Indian standards, the permissible phenol concentration in drinking water should be limited to 0.002 mg/L [14]. However, due to its intrinsic chemical stability, phenol is highly resistant to conventional treatment technologies, such as biological degradation and physical adsorption, leading to its long-term persistence in the environment [15]. Traditional approaches, including solvent extraction, adsorption, and enzymatic catalysis, also suffer from significant limitations: solvent extraction often causes secondary pollution, adsorbents such as activated carbon are expensive and have limited reusability, and enzyme-based methods are hindered by poor stability and short operational lifetimes [16,17]. These drawbacks highlight the urgent need for more efficient, economical, and environmentally sustainable technologies for phenol removal.

Among various advanced oxidation processes (AOPs), the Fenton reaction has attracted extensive attention due to its strong oxidative capability, operational simplicity, and proven effectiveness in degrading refractory organic pollutants [18]. It has demonstrated particularly promising potential for industrial applications, particularly for treating phenolic wastewater. During Fenton and Fenton-like oxidation of phenol, multiple intermediate products are typically formed. In a simplified pathway, phenol is first oxidized to intermediates, such as catechol, resorcinol, and hydroquinone, followed by further oxidation into mineralized end products, primarily CO_2_ and H_2_O [19,20]. In recent years, Fe-based amorphous alloys have emerged as a new class of high-performance catalysts for AOPs [21]. Their atomic-level disordered structure, metastable nature, and abundance of catalytically active sites enable rapid electron transfer and homogeneous reaction interfaces [22]. Compared with crystalline iron-based materials, Fe-based amorphous alloys exhibit higher reactivity and better corrosion resistance, making them highly attractive for Fenton-like catalysis and environmental remediations [23]. For example, Wang et al. [24] reported that Fe_78_Si_9_B_13_ amorphous alloy exhibited promising catalytic activity toward phenol degradation, with the reaction primarily occurring at the interface between zero-valent iron (ZVI) and water. The formation and evaluation of surface oxide layers were found to play a crucial role in controlling the degradation rate [25]. Similarly, Ni et al. [26] prepared nano-porous Fe_76_Si_9.6_B_7.2_P_7.2_ amorphous alloys via dealloying and achieved superior catalytic efficiency owing to their high surface area and uniform distribution of active iron sites.

Recent advances in atomic-scale manufacturing strategies have enabled precise control over the surface structure and chemical composition of Fe-based amorphous alloys [27,28]. In particular, the construction of Fe-Cu galvanic couples on amorphous alloy surfaces through controlled atomic substitution or surface co-deposition can create localized galvanic interactions [29]. These interactions facilitate continuous Fe^0^/Fe^2+^ regeneration and accelerate the decomposition of hydrogen peroxide, thereby promoting the generation of reactive hydroxyl radicals (·OH) [30].

In this study, a novel atomic-scale fabrication approach was developed to introduce micro/nano Fe-Cu galvanic couples on Fe-based amorphous alloy. Fe_73.5_Si_13.5_B_9_Cu_1_Nb_3_ amorphous alloy ribbons were selected as model catalysts to investigate the influence of these micro/nano Fe-Cu galvanic structures on phenol degradation performance in a Fenton-like reaction system. A simulated coal-chemical wastewater containing 50 mg/L phenol was used to evaluate the degradation behavior under various environmental conditions, including temperature, pH, hydrogen peroxide concentration, and catalyst dosage. This study aims to demonstrate the feasibility of enhancing the phenol degradation performance of Fe-based amorphous alloys through atomic-scale surface engineering and to provide a new pathway for the efficient removal of refractory organic pollutants. Additionally, the plausible degradation mechanism of phenol over the Fe_73.5_Si_13.5_B_9_Cu_1_Nb_3_ amorphous alloy was discussed to gain deeper insight into its structure-activity relationship.

## 2. Materials and Methods

### 2.1. Materials and Fabrication of Micro/Nano Fe-Cu Couples

Phenol (C_6_H_6_O, AR) was purchased from Shanghai Aladdin Bio-Chem Technology Co., Ltd. (Shanghai, China). Sulfuric acid (H_2_SO_4_, 98 wt.%, AR) was obtained from Shanghai Titan Technology Co., Ltd. (Shanghai, China), while hydrogen peroxide (H_2_O_2_, 30 wt.%) and copper sulfate (CuSO_4_, ≥99.0%) were purchased from Tianjin Yongda Chemical Reagent Co., Ltd. (Tianjin, China). All chemicals were of analytical grade and used without further purification. Deionized water (resistivity < 5 μS/cm) was used throughout all experiments for solution preparation and dilution.

Fe-based amorphous alloy ribbons with a nominal composition of Fe_73.5_Si_13.5_B_9_Cu_1_Nb_3_ (at. %) were purchased from Jiangsu Jicui Antai Chuangming Advanced Energy Materials Research Institute Co., Ltd. (Changzhou, China). The as-cast Fe-based amorphous alloy ribbons, with an average thickness of 25 μm and a width of 8 mm, were cut into 10–20 mm pieces prior to use. The micro/nano Fe-Cu galvanic couples were fabricated on the surface of Fe-based amorphous ribbons via a two-step chemical treatment. First, a precursor solution (denoted as PC × 1) was prepared by dissolving CuSO_4_ and H_2_SO_4_ in deionized water to achieve Cu^2+^ and SO_4_^2−^ concentrations of 6.25 mmol/L and 12.5 mmol/L, respectively. For PC × 2 or PC × 4, the concentration of ions will be doubled or quadrupled, respectively. Subsequently, the Fe_73.5_Si_13.5_B_9_Cu_1_Nb_3_ ribbons were immersed in the precursor solution for 15 s to induce an in situ redox reaction between Fe^0^ and Cu^2+^ ions, resulting in the spontaneous formation of micro/nano Fe-Cu galvanic couples. After immersion, the samples were ultrasonically rinsed with deionized water for 1 min to remove any loosely adhered species. The as-prepared Fe-based amorphous alloys with micro/nano Fe-Cu galvanic couples were then dried at room temperature and used for subsequent catalytic degradation towards phenol.

### 2.2. Microstructure Characterization

The crystalline structure and phase composition of the Fe-based amorphous alloy ribbons were characterized by X-ray diffraction (XRD) using a DX-2700B diffractometer (Dandong Haoyuan Instrument Co., Ltd., Dandong, China). Diffraction patterns were collected using Cu Kα radiation (λ = 1.5406 Å) over a 2θ range of 20–90°, with a step interval of 0.02° and a total acquisition time of at least 15 min per sample. The surface morphology of the samples was examined using a scanning electron microscope (SEM, TESCAN, GAIA3, Brno, Czech Republic), equipped with an energy-dispersive X-ray spectroscopy (EDS, OXFORD instruments, Ultim-Max, Abingdon, UK) system for elemental analysis. The SEM–EDS combination enabled simultaneous observation of surface microstructures and determination of the corresponding elemental distribution in selected micro regions. X-ray photoelectron spectroscopy with Al-Kα radiation at room temperature (XPS, ESCALAB 250Xi, Thermo Scientific, MA, USA) investigated the valence states of the main elements’ valence states.

### 2.3. Phenol Degradation Experiments

**Figure 1:**
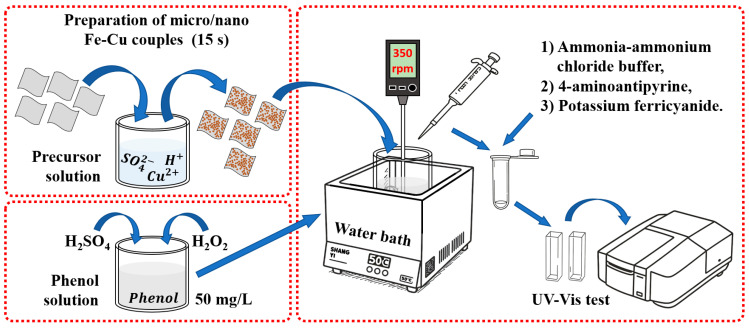
Schematic illustration of the fabrication of micro/nano Fe-Cu galvanic couples on the Fe-based amorphous alloy, followed by their application in the phenol degradation experiment.

For the phenol degradation experiments, 250 mL of phenol solution (50 mg/L) was prepared to simulate coal chemical wastewater. Commercially available hydrogen peroxide (H_2_O_2_) was employed as the oxidizing agent and added externally at predetermined concentrations (0–20 mmol/L). The solution pH was monitored using a benchtop pH meter (LC-PH-2B, Lichen Technology, Shaoxing, China) and adjusted to the desired values using either 0.1 mol/L NaOH or 5 wt.% dilute H_2_SO_4_. The optimized pH condition for most Fenton reactions is approximately 3.0, which requires ~50 g of 98 wt.% H_2_SO_4_ to adjust 1 ton of water to pH 3; the corresponding cost is approximately USD $0.14 per ton, based on pricing information from Nanjing Reagent (Nanjing, China). The reaction temperature was maintained at 323 K using a thermostatic water bath. The Fe_73.5_Si_13.5_B_9_Cu_1_Nb_3_ amorphous alloy ribbons were cut into 1~2 cm segments and added to the phenol solution at the designed dosage to initiate the degradation reactions. The suspension was mechanically stirred throughout the reaction to ensure adequate contact between the alloy and the solution. During the degradation process, 0.25 mL aliquots were withdrawn every 0.5–3 min for concentration analysis. A schematic illustration of the fabrication of the micro/nano Fe-Cu galvanic couples and the phenol degradation process is shown in Figure 1.

**Figure 2:**
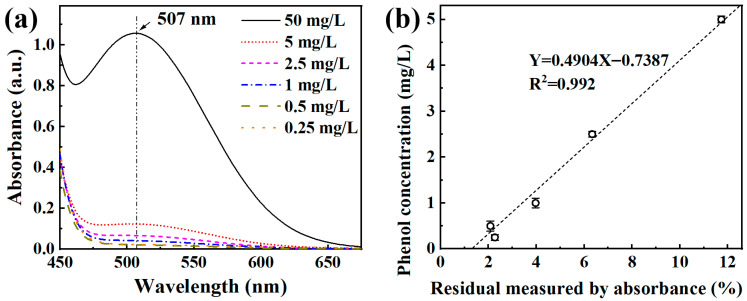
Concentration of phenol solution and its absorbance (**a**) UV spectrum and (**b**) phenol concentration vs. residual UV-vis absorbance fitting curve.

Phenol concentrations were determined using the 4-aminoantipyrine colorimetric method. Each collected sample was sequentially mixed with 0.05 mL of 20 wt.% ammonia-ammonium chloride buffer, 0.1 mL of 2 wt.% 4-aminoantipyrine solution, and 0.1 mL of 8 wt.% potassium ferricyanide solution. After thorough mixing, the solution was diluted to 4 mL with deionized water and analyzed using a UV–Vis spectrophotometer at 507 nm, with deionized water as the reference. A standard calibration curve was established by measuring the absorbance of phenol solutions diluted from 50 mg/L to 10%, 5%, 2%, 1%, and 0.5% of the initial concentration, yielding a linear relationship between absorbance and phenol concentration, as shown in Figure 2.

Based on the established calibration curve, the phenol removal efficiency during the degradation was quantified using Equation (1). The degradation kinetic of the system were analyzed according to a pseudo-first-order kinetic model, and the degradation rate constant *k_obs_* was determined using Equation (2) [31]. To facilitate a clear comparison of phenol degradation efficiency and the corresponding ln(C_0_/Cₜ) curves under different experimental conditions, only representative results were selected for each set of conditions. For the comparison of *k_obs_* values in the Section 4, a minimum of three independent experiments were conducted for each condition to obtain the average rate constant and the associated error bars.

(1) $$Phenol\;removal=\left(C_{0}-C_{t}\right)/C_{0}\times 100$$

(2) $$ln\left(C_{0}/C_{t}\right)=k_{obs}t_{d}$$

where $C_{0}$ is the initial concentration of phenol (mg/L); $C_{t}$ is the concentration of phenol at t minutes of degradation; $t_{d}$ is the degradation time; $k_{obs}$ is the degradation constant.

## 3. Results

### 3.1. The Intrinsic Catalytic Performance of Fe_73.5_Si_13.5_B_9_Cu_1_Nb_3_

**Figure 3:**
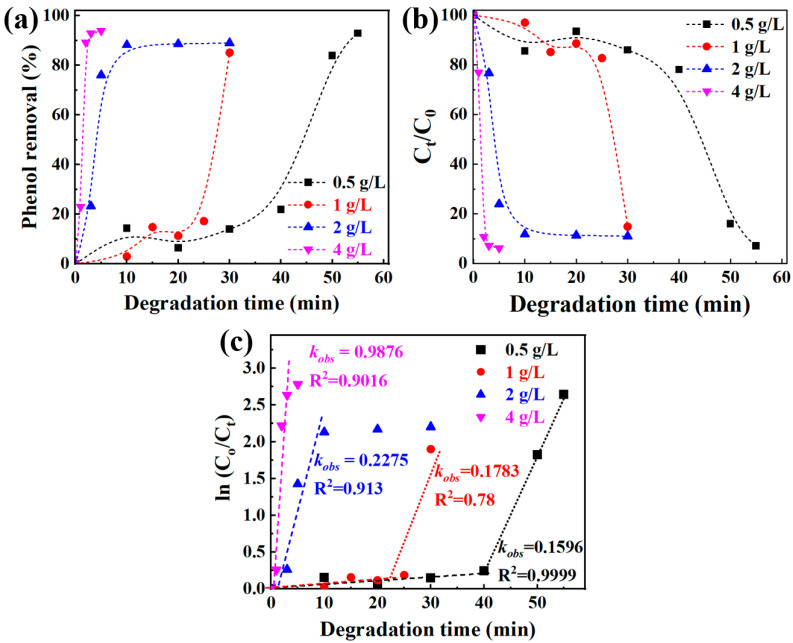
Phenol degradation by Fe_73.5_Si_13.5_B_9_Cu_1_Nb_3_ amorphous alloy with different dosages, typical plots of (**a**) phenol removal vs. degradation time, (**b**) C_t_/C_0_ vs. degradation time, and (**c**) ln(C_0_/C_t_) vs. degradation time.

To evaluate the intrinsic catalytic performance of Fe_73.5_Si_13.5_B_9_Cu_1_Nb_3_ amorphous alloy, the influence of catalyst dosage on the degradation efficiency of phenol was investigated. The experiments were conducted at 50 °C, pH = 3, with an initial phenol concentration (C_0_) of 50 mg/L, and H_2_O_2_ concentration of 10 mmol/L. These parameters were selected based on a series of preliminary optimization tests. The catalyst dosages were varied at 0.5, 1, 2, and 4 g/L, which are equal to 25.8, 51.6, 103.3, and 206.6 mmol/L, respectively. The corresponding degradation behaviors are presented in Figure 3.

The catalytic degradation efficiency of phenol increased progressively with increasing catalyst loading. When the dosage was 25.8 mmol/L, the Fe_73.5_Si_13.5_B_9_Cu_1_Nb_3_ amorphous alloy achieved 92.9% phenol removal within 55 min, corresponding to an observed rate constant (*k_obs_*) of 0.1596 min^−1^. Increasing the dosage to 51.6 mmol/L shortened the degradation time to 30 min, although the removal efficiency slightly decreased to 85.1%, with a marginally higher rate constant of 0.1783 min^−1^. Notably, an induction period was observed when the catalyst dosage was less than 51.6 mmol/L, suggesting an initial activation process of the amorphous alloy surface prior to the generation of reactive species.

A pronounced enhancement in catalytic activity was observed when the dosage reached 103.3 mmol/L. Under this condition, near-complete phenol degradation was achieved within 10 min, with a significantly higher rate constant of 0.2275 min^−1^. Further increasing the dosage to 206.6 mmol/L led to complete degradation within 5 min, achieving a phenol removal efficiency of 93.8% and a significantly higher rate constant of 0.9876 min^−1^. At dosages higher than 103.3 mmol/L, the induction period disappeared, indicating a large number of active sites available to rapidly initiate the Fenton-like reactions. These results clearly demonstrate that the catalyst dosage plays a decisive role in determining the degradation efficiency. Increasing the alloy loading enhances both the degradation rate and overall removal efficiency, resulting in faster and more complete phenol decomposition. The observed trend can be attributed to the increased number of active surface sites and larger interfacial area provided by the higher catalyst dosage, which promotes the generation of hydroxyl radicals (·OH) through Fenton-like reactions, as described in Equations (3) and (4). Since ·OH serve as the dominant oxidizing species in this system, their elevated concentration directly enhances the degradation kinetics of phenol, according to Equations (5) and (6) [29,32]. Moreover, because the establishment of a multivalent iron ion system requires a longer time when a lower dosage of the Fe-based catalyst is used, the activation process observed in Figure 3 can be attributed to the generation of Fe^2+^ and Fe^3+^ from Fe^0^, as described in Equations (3)–(5).

(3) $${Fe}_{solid}^{0}+2H^{+}\to {Fe}_{aq}^{2+}+H_{2}↑$$

(4) $${Fe}_{aq}^{2+}+H_{2}O_{2}+H^{+}\to {Fe}_{aq}^{3+}+H_{2}O+\cdot OH$$

(5) $$2{Fe}_{aq}^{3+}+{Fe}_{solid}^{0}\to 3{Fe}_{aq}^{2+}$$

(6) ·OH+phenol→degradation products

**Figure 4:**
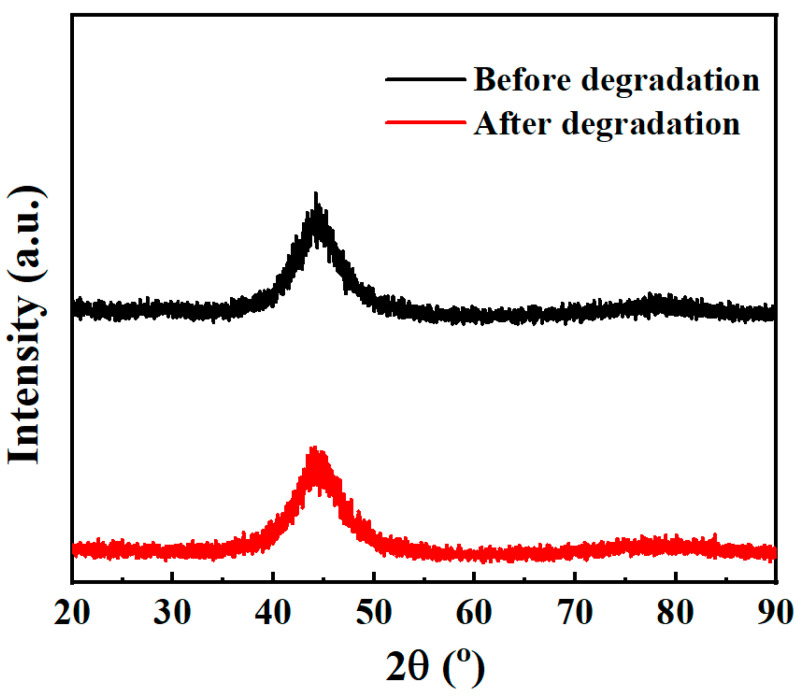
Phase analysis of Fe_73.5_Si_13.5_B_9_Cu_1_Nb_3_ amorphous alloy by XRD.

**Figure 5:**
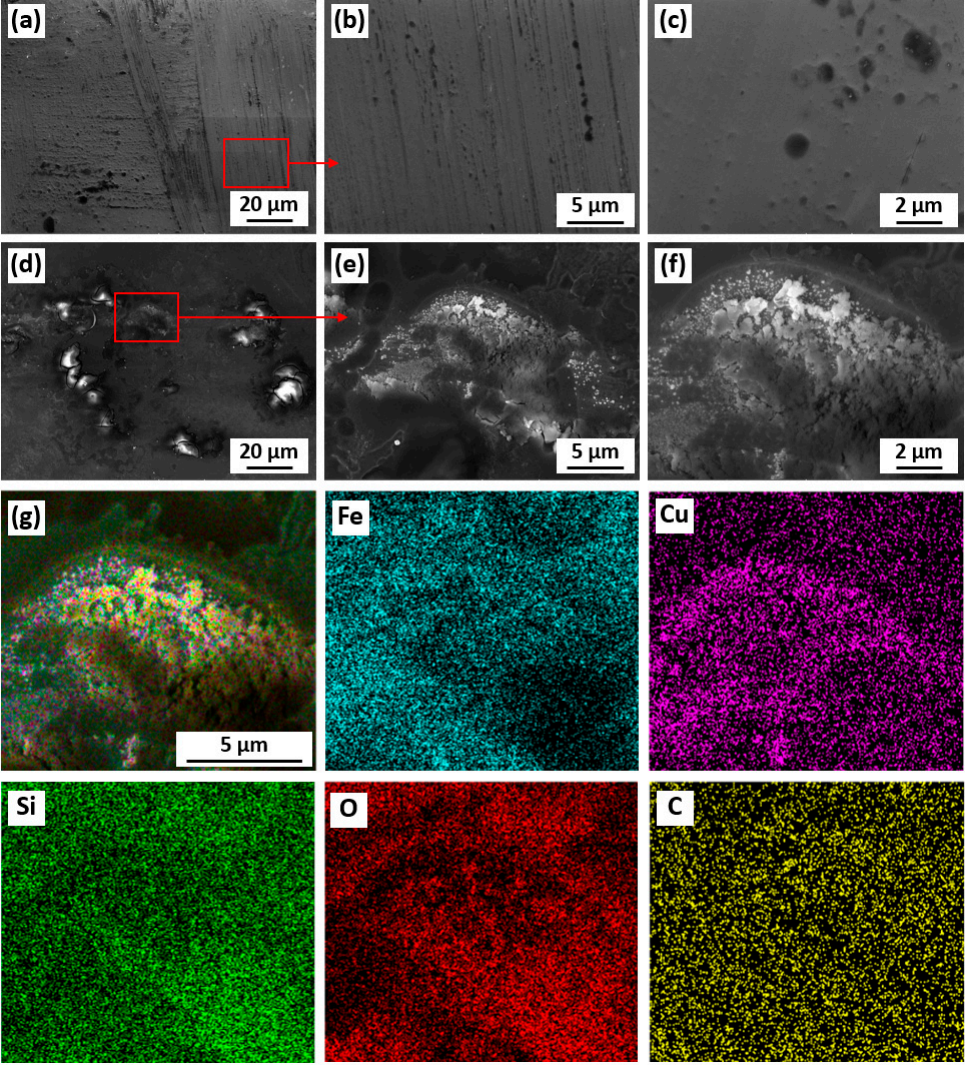
Microstructure and morphology of Fe_73.5_Si_13.5_B_9_Cu_1_Nb_3_ amorphous alloy surface (**a**–**c**) before and (**d**–**f**) after degradation of phenol, and (**g**) EDS analysis of the Fe_73.5_Si_13.5_B_9_Cu_1_Nb_3_ amorphous alloy after phenol degradation, combined with EDS maps of Fe, Cu, Si, O, and C.

To elucidate the underlying mechanism responsible for the superior catalytic activity of the Fe_73.5_Si_13.5_B_9_Cu_1_Nb_3_ amorphous alloy, its microstructure, phase composition, and surface morphology before and after reaction were systematically examined (Figure 4 and Figure 5 and Table 1). The outstanding catalytic performance can be attributed to the alloy’s metastable amorphous structure, which provides abundant, uniformly distributed reactive centers for the Fenton-like interaction with H_2_O_2_, thereby facilitating efficient phenol degradation.

As shown in Figure 4, the XRD patterns of the Fe_73.5_Si_13.5_B_9_Cu_1_Nb_3_ amorphous alloy before and after the degradation reaction exhibit similar broad diffuse halos without any sharp crystalline diffraction peaks, confirming the fully amorphous nature of the material. Although slight oxidation may occur during the degradation process, no new crystallization peaks were detected, indicating that any crystal-structural changes are negligible. After phenol degradation, the Fe_73.5_Si_13.5_B_9_Cu_1_Nb_3_ alloy clearly maintained its amorphous state, demonstrating excellent amorphous stability during the degradation reactions. Given that phenol degradation primarily occurs at the solid–liquid interface, the surface morphology of the alloy was further examined using SEM (Figure 5). Before the reaction (Figure 5a–c), the alloy exhibited a smooth and homogeneous surface without visible cracks or corrosion features. After phenol degradation (Figure 5d–f), significant morphological changes were observed, which includes the generation of abundant microcracks and reaction-product deposits distributed across the corroded regions. SEM images at higher magnifications further revealed a densely stacked lamellar structure on the alloy surface, which might benefit the adsorption of organic molecules and the reaction between the water-soluble substances and metallic active sites. This microstructural evolution facilitates the continuous generation of hydroxyl radicals, thereby accelerating the catalytic degradation of phenol.

The EDS results (Table 1 and Figure 5g) further elucidate the compositional evolution of the Fe_73.5_Si_13.5_B_9_Cu_1_Nb_3_ amorphous alloy during the catalytic degradation process. The pristine alloy surface consisted of 84.05 at. % Fe, 5.13 at. % Nb, and 1.97 at. % Cu. After the degradation reaction, the Fe content markedly decreased to 41.92 at. %, accompanied by a reduction in Nb content to 0.28 at. %. The decrease in Fe content indicates the leaching of iron ions, which was confirmed by ICP measurements. The Fe concentration in the treated phenol solution ranged from 1 to 8 mg/L, depending on the degradation conditions and catalyst dosage. In contrast, the O and Cu contents increased to 16.0 at. % and 8.06 at. %, respectively. These compositional variations demonstrate that surface oxidation occurred during the degradation process, resulting in the consumption of ZVI, which acts as the anode and electron donor, as illustrated in Figure 5. The ZVI releases electrons that are subsequently transferred to adjacent metal atoms or reactive oxygen intermediates, thereby promoting the generation of reactive species responsible for phenol oxidation. The increase in oxygen content combined with the high-resolution XPS spectra of Fe 2p, Si 2p, and O 1s (exhibited in Section 3.3) signifies the formation of Fe- or Si-based oxides on the alloy surface, which can act as adsorption and reaction sites for intermediates involved in the Fenton-like process. Meanwhile, the enrichment of copper, along with its elemental distribution map that differs from oxygen, suggests that copper predominantly remained in a metallic state. This metallic copper acts as a cathode site, accelerating electron transfer within the Fe/Cu micro-galvanic system and thereby enhancing the overall redox activity. Niobium, on the other hand, plays a stabilizing role in maintaining the amorphous structure by hindering atomic diffusion and crystallization. The observed decrease in niobium content after reaction implies partial degradation or coverage of the Nb-enriched surface layer, possibly due to the formation of iron oxides or deposition of organic intermediates during the reaction.

Collectively, the microstructural, morphological, and compositional analyses confirm that the high catalytic effectivity of the Fe_73.5_Si_13.5_B_9_Cu_1_Nb_3_ amorphous alloy originates from the synergistic effects of its metastable amorphous structure and the in situ formation of Fe-Cu micro-galvanic couples. The disordered atomic arrangement and multielement composition enable rapid Fe^2+^/Fe^3+^ redox cycling and efficient hydroxyl radical generation, while the robust amorphous matrix ensures high structural stability and sustained activity throughout the degradation process. These findings provide new insights into the catalytic mechanism and offer a potential pathway for further improving the degradation performance of Fe-base amorphous alloys in Fenton-like systems.

### 3.2. The Effect of Environmental Factors on Degradation of Phenol

In practical wastewater treatment systems, parameters such as temperature, pH, hydrogen peroxide dosage, and catalyst loading play critical roles in determining the overall treatment efficiency. Considering their importance, these environmental factors were systematically investigated to evaluate the catalytic degradation performance of Fe_73.5_Si_13.5_B_9_Cu_1_Nb_3_ amorphous alloy toward phenol. To ensure consistency and comparability, Fe_73.5_Si_13.5_B_9_Cu_1_Nb_3_ amorphous alloys were uniformly employed as the catalyst throughout all experiments.

- Effect of H_2_O_2_ concentration on phenol degradation

**Figure 6:**
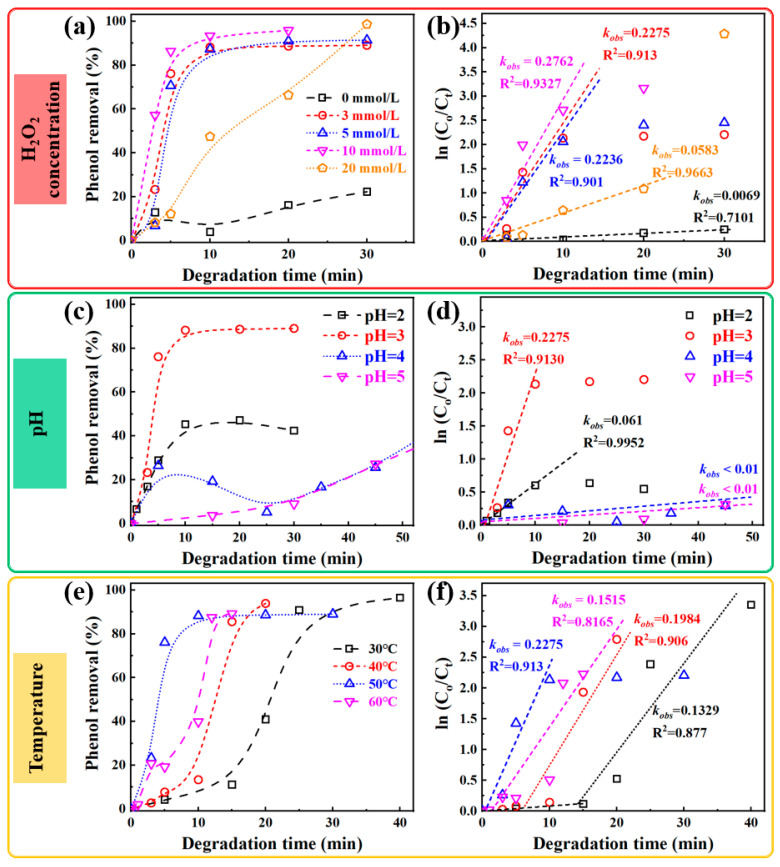
Degradation of phenol by Fe_73.5_Si_13.5_B_9_Cu_1_Nb_3_ amorphous alloy at different conditions, (**a**,**b**) H_2_O_2_ concentration, (**c**,**d**) pH, and (**e**,**f**) temperature. (**a**,**c**,**d**) typical plots of phenol removal vs. degradation time, and (**b**,**d**,**f**) typical plots of ln(C_0_/C_t_) vs. degradation time.

In the phenol degradation using Fe_73.5_Si_13.5_B_9_Cu_1_Nb_3_ amorphous alloy, the concentration of hydrogen peroxide is a key factor influencing the generation rate of hydroxyl radicals (·OH) in Fenton-like systems. To elucidate the effect of H_2_O_2_ concentration on the catalytic performance of Fe_73.5_Si_13.5_B_9_Cu_1_Nb_3_ amorphous alloy toward phenol degradation, experiments were conducted under controlled conditions: temperature = 50 °C, catalyst dosage = 2 g/L, initial phenol concentration (C_0_) = 50 mg/L, and pH = 3. Different H_2_O_2_ concentrations (0, 3, 5, 10, and 20 mmol/L) were added to the phenol solution, and the degradation behaviors were monitored accordingly. The corresponding degradation profiles are presented in Figure 6a,b.

As shown in Figure 6a, the phenol removal rate was strongly dependent on the H_2_O_2_ concentration. In the absence of H_2_O_2_, the Fe_73.5_Si_13.5_B_9_Cu_1_Nb_3_ amorphous alloy exhibited very limited activity, achieving only 22.3% decolorization within 30 min with a reaction rate constant (*k_obs_*) of 6.9 × 10^−3^ min^−1^. This result indicates that H_2_O_2_ is indispensable for ·OH generation and effective phenol degradation. Upon increasing the H_2_O_2_ concentration to 3 mmol/L, the phenol degradation efficiency improved remarkably ~89% within 30 min, and *k_obs_* increased to 0.2236 min^−1^. This enhancement demonstrates that moderate H_2_O_2_ addition significantly accelerates the Fenton-like reaction, thereby promoting the production of ·OH.

When the H_2_O_2_ concentration was further raised to 5–10 mmol/L, the catalytic degradation efficiency continued to increase. At 5 and 10 mmol/L, phenol was almost completely degraded within 20 min, with decolorization efficiencies of 91.4% and 98.6%, while the reaction rate constants remain the same level, i.e., 0.2236 (for 5 mmol/L) and 0.2762 (for 10 mmol/L) min^−1^. These results suggest that increasing H_2_O_2_ concentration enhances ·OH generation within a certain range, thereby significantly improving the catalytic efficiency of this Fe-based amorphous alloy. However, excessive H_2_O_2_ concentration adversely affected the degradation performance. When the concentration reached 20 mmol/L, the phenol removal could reach a high level of 98.6%, but the reaction rate constant within the 20 min reaction decreased to 0.0583 min^−1^. This decline might be attributed to the scavenging effect of excess H_2_O_2_, where ·OH could be consumed by H_2_O_2_ itself or iron ions, according to the following side reaction, Equations (7) and (8) [29],

(7) $$\cdot OH+H_{2}O_{2}\to HO_{2}^{\cdot }+H_{2}O$$

(8) $${Fe}^{3+}+H_{2}O_{2}\to {Fe}^{2+}+HO_{2}^{\cdot }+H^{+}$$

This self-quenching process reduces the effective ·OH concentration, thereby suppressing the overall degradation rate. Therefore, an optimal H_2_O_2_ concentration is essential to balance radical generation and scavenging and thereby maximize catalytic efficiency.

- Effect of pH on phenol degradation

According to Equation (4), the effect of pH is closely to that of hydrogen peroxide, as variations in pH directly determine the concentration of H^+^, which plays a crucial role in governing the degradation efficiency. To isolate the influence of pH, all other reaction parameters were kept constant: temperature = 50 °C, catalyst dosage = 2 g/L, initial phenol concentration (C_0_) = 50 mg/L, and hydrogen peroxide concentration = 10 mmol/L. The phenol removal efficiency and the corresponding kinetic constant *k_obs_* at different pH values are shown in Figure 6c,d.

At pH = 2, the Fe_73.5_Si_13.5_B_9_Cu_1_Nb_3_ amorphous alloy exhibited relatively low activity, removing only 42.3%, of phenol within 30 min, with a reaction rate constant of 0.061 min^−1^. When the pH increased to 3, the degradation performance improved remarkably, achieving 89% removal of phenol within 10 min and a kinetic constant of 0.2275 min^−1^. However, further increases in pH led to a sharp decline. At pH = 4 and 5, only ~40% of phenol was decomposed within 50 min, with a removal efficiency of 40% and *k_obs_* < 0.01 min^−1^, which illustrated that the phenol degradation efficiency of Fe_73.5_Si_13.5_B_9_Cu_1_Nb_3_ amorphous alloy increases with pH up to an optimum value of 3 and decreases thereafter.

The dependence of phenol degradation on pH can be attributed to multiple physicochemical factors. Under acidic conditions, the ZVI at the Fe-based amorphous alloy surface reacts with H^+^ according to Equation (3), generating Fe^2+^ ions, which subsequently react with H_2_O_2_ to produce hydroxyl radicals (·OH) and Fe^3+^ Ions following Equation (4) [33]. Moreover, in acidic media, Fe^3+^ ions can be reduced back to Fe^2+^ either by ZVI or through Fenton-like pathways (Equations (5) and (8)), sustaining the Fe^2+^/Fe^3+^ redox cycle and promoting continuous hydroxyl radicals (·OH) generation.

However, when the pH becomes excessively low, the excessive proton concentration intensifies hydrogen evolution, forming numerous tiny hydrogen bubble that accumulate on the catalyst surface and hinder contact between the alloy and the solution. In addition, the excess H^+^ promotes the scavenging of hydroxyl radicals and Fe^2+^ ions, converting them into Fe^3+^ and water (Equation (9)), which suppresses hydroxyl radicals concentration and weakens the Fe^2+^/Fe^3+^ redox cycle, ultimately lowering the degradation efficiency of Fe-based amorphous alloys.

(9) $${Fe}_{aq}^{2+}+\cdot OH+H^{+}\to {Fe}_{aq}^{3+}+H_{2}O$$

Under neutral or alkaline conditions, the ZVI on the surface of the Fe_73.5_Si_13.5_B_9_Cu_1_Nb_3_ amorphous alloy has difficulty reacting simultaneously with H+ and H_2_O_2_. As a result, only a small amount of Fe^2+^ can be generated from ZVI, and the available Fe^2+^ is more readily oxidized to Fe^3+^, as described in Equation (10). In alkaline media, both Fe^2+^ and Fe^3+^ tend to form their corresponding hydroxides according to Equations (11) and (12), leading to the accumulation of oxide or hydroxide layers on the alloy surface [33]. This passivation layer hinder electron transfer and suppress the catalytic decomposition of H_2_O_2_ into ·OH due to the insufficient availability of free Fe^2+^/Fe^3+^ ions in the solution.

(10) $${Fe}^{2+}+H_{2}O_{2}\to {Fe}^{3+}+\cdot OH+{OH}^{-}$$

(11) $${Fe}^{3+}+3{OH}^{-}\to Fe{\left(OH\right)}_{3}↓$$

(12) $${Fe}^{2+}+2{OH}^{-}\to Fe{\left(OH\right)}_{2}↓$$

Consequently, the alloy gradually loses its catalytic activity, and the phenol degradation efficiency is dramatically reduced. Therefore, maintaining a mildly acidic environment (pH ≈ 3) is essential for achieving optimal phenol degradation performance using the Fe_73.5_Si_13.5_B_9_Cu_1_Nb_3_ amorphous alloy.

- Effect of reaction temperature on phenol degradation

To examine the influence of temperature, degradation experiments were conducted under controlled conditions, with a catalyst dosage of 2 g/L, initial phenol concentration (C_0_) of 50 mg/L, pH = 3, and hydrogen peroxide concentration of 10 mmol/L. The degradation efficiency of phenol at different reaction temperatures is illustrated in Figure 6e,f. It can be seen that Fe_73.5_Si_13.5_B_9_Cu_1_Nb_3_ amorphous alloy exhibited acceptable degradation performance at 30 °C, achieving nearly complete degradation of phenol within 40 min, with a kinetic constant of 0.1329 min^−1^. Increasing the temperature to 40 °C enhanced the degradation rate, enabling complete removal of phenol within 20 min, with the rate constant increasing to 0.1984 min^−1^ and a phenol removal efficiency of 93.8%. When the temperature was further elevated to 50 °C, the alloy maintained superior catalytic performance, degrading most phenol within 10 min, corresponding to a rate constant of 0.2275 min^−1^ and decolorization efficiency of 89%. However, further temperature elevation to 60 °C will led to a decline, with the rate constant dropping to 0.1515 min^−1^ and phenol removal of 89% in 15 min, which demonstrates that the catalytic efficiency of Fe_73.5_Si_13.5_B_9_Cu_1_Nb_3_ amorphous alloy increases with temperature initially but decreases beyond 50 °C, where the optimal catalytic performance is achieved.

The variation in degradation performance of Fe_73.5_Si_13.5_B_9_Cu_1_Nb_3_ amorphous alloy could be explained using the Arrhenius relation. As the temperature rises, the formation rate of hydroxyl radicals (OH) accelerates, and the average kinetic energy of reactant molecules increases, leading to an enhanced reaction rate constant and accelerating the degradation performance, which is consistent with previous reports [33,34]. However, when the temperature exceeds a critical threshold, thermal decomposition of H_2_O_2_ becomes dominant, resulting in a reduced ·OH yield. Consequently, the overall degradation efficiency and phenol removal rate decrease in this temperature range. Therefore, in practical applications, the reaction temperature must be carefully optimized to ensure the best phenol degradation performance.

**Figure 7:**
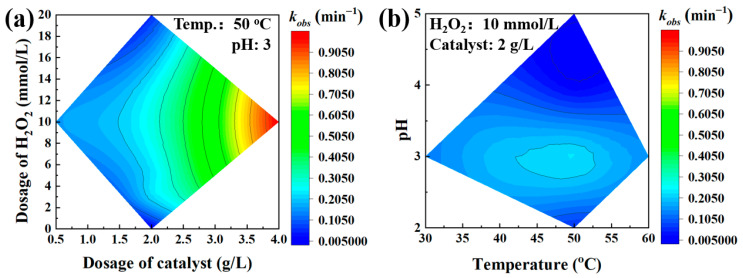
Contour maps of phenol degradation rate constant (*k_obs_*) by Fe_73.5_Si_13.5_B_9_Cu_1_Nb_3_ amorphous alloy as a function of (**a**) dosage of H_2_O_2_ and catalyst and (**b**) pH and degradation temperature.

Based on the above investigation results, contour map of kinetic constant as a function of H_2_O_2_ concentration and the dosage of the Fe-based alloy was generated and shown in Figure 7a. This contour illustrate that a dosage of catalyst increases from 0.5 g/L to 4 g/L significantly improved the degradation rate constant. Figure 7b exhibits the contour map of kinetic constant as a function of pH and reaction temperature, which discloses the optimization of pH and temperature for this Fenton-like reaction system are 3 and 50 °C, respectively. Combine these two figures together, it can be seen that the dosage of Fe_73.5_Si_13.5_B_9_Cu_1_Nb_3_ amorphous alloy dominates the phenol degradation rate; however, a high consumption level of catalyst and H_2_O_2_ makes the high cost and low economic efficiency. Therefore, it is important to improve the catalytic activity and lower the consuming of the Fe-based amorphous alloy.

Here, the optimized environmental conditions for phenol degradation using Fe_73.5_Si_13.5_B_9_Cu_1_Nb_3_ amorphous alloy are: temperature of 50 °C and pH = 3. As for H_2_O_2_, it shows a slight influence of on the phenol degradation rate, when its concentration ranges from 3 to 15 mmol/L. Thus, a relative low concentration, i.e., 3 mmol/L, is picked for the following investigation to meet the cost-effective target.

### 3.3. The Fabrication of Micro/Nano Fe-Cu Galvanic Couples and Its Effect on Phenol Degradation

**Figure 8:**
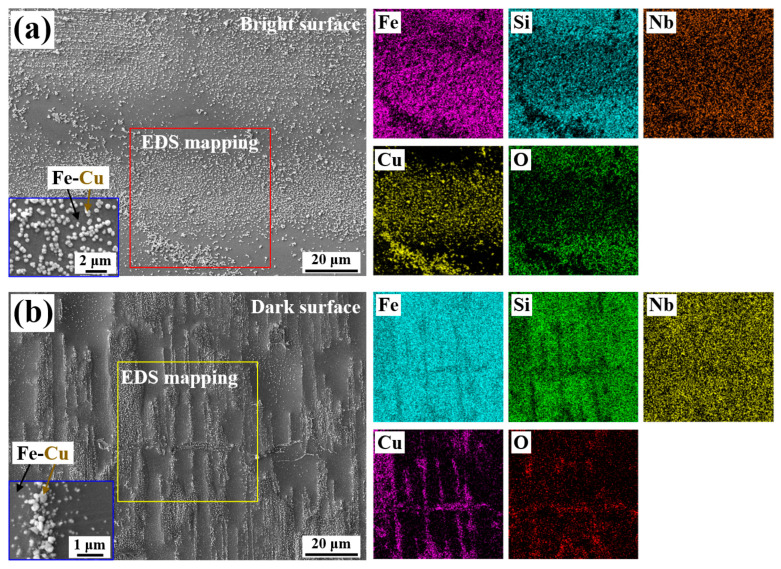
Typical microstructure and morphology of the as-fabricated Fe-Cu galvanic couples on Fe_73.5_Si_13.5_B_9_Cu_1_Nb_3_ amorphous alloy’s (**a**) bright surface and (**b**) dark surface, combined with EDS maps of Fe, Si, Nb, Cu, and O. The inserted SEM images with higher magnifications exhibit the Fe-Cu couples.

Figure 8 presents the typical microstructure of the micro/nano Fe-Cu galvanic couples fabricated on the Fe_73.5_Si_13.5_B_9_Cu_1_Nb_3_ amorphous alloy surface, and the precursor concentration used for this sample is PC × 1, which contained Cu^2+^ and SO_4_^2−^ with concentrations of 6.25 × 10^−3^ mol/L and 1.25 × 10^−2^ mol/L, respectively. The Fe-Cu couples show a uniform particle size, less than 5 µm. Because Fe-based amorphous ribbons produced by melt-spinning inherently possess two distinct surfaces, both the bright side (smooth and reflective) and the dark side (rougher and containing micro-textures) were examined using SEM. Notably, the two sides of the Fe_73.5_Si_13.5_B_9_Cu_1_Nb_3_ amorphous alloy ribbon display different distributions of Fe-Cu couples.

By comparing Figure 8a,b, a distinct difference in surface morphology was observed between the bright surface and dark surfaces of the Fe-based amorphous alloy ribbons. The bright side exhibited a relatively smooth and uniform surface, whereas the dark side showed a more undulated topography. Also, the micro/nano Fe-Cu couples generated on the bright surface are more uniform and well-distributed than those formed on the dark surface. As shown in Figure 8b, the areas covered by Fe-Cu galvanic couples in the dark surface were predominantly distributed at the “highland” regions of the surface, while the lower-lying regions tended to remain as bare areas without Fe-Cu deposition.

According to the EDS analysis, the recessed regions on the dark surface contained a higher concentration of Si, which suggests that during the reaction between the Fe-based amorphous alloy and the precursor solution, Si-enriched regions were less reactive toward Cu^2+^ ions, thereby inhibiting the substitution reaction necessary for Fe-Cu couple formation. A similar tendency was also observed on the bright surface of the alloy, where areas with locally high Si content exhibited a noticeably lower density of Fe-Cu couples compared to the average distribution across the surface. These findings indicate that the local surface composition, particularly Si enrichment, plays a critical role in governing the formation and spatial distribution of Fe-Cu galvanic couples on the Fe-based amorphous alloy surface.

**Figure 9:**
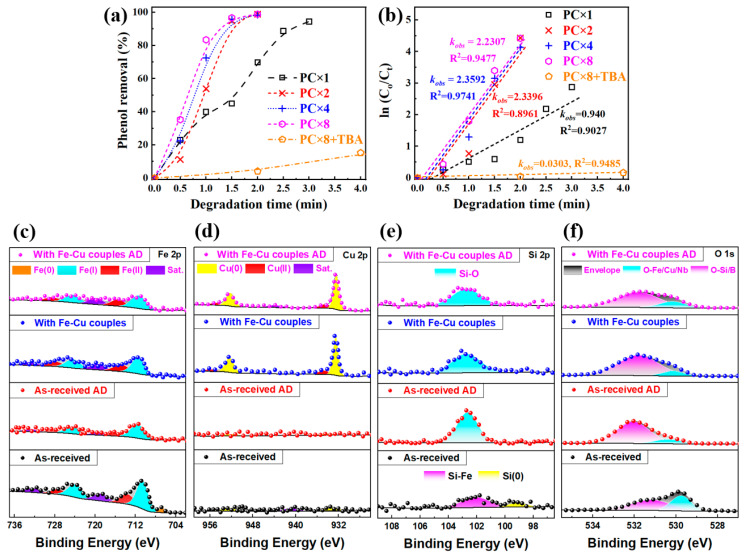
Degradation of phenol by Fe_73.5_Si_13.5_B_9_Cu_1_Nb_3_ amorphous alloy with micro/nano Fe-Cu galvanic couples, prepared by different precursor concentrations, typical plots of (**a**) phenol removal vs. degradation time, and (**b**) In(C_0_/C_t_) vs. degradation time. High-resolution of XPS spectra of (**c**) Fe 2p, (**d**) Cu 2p, (**e**) O 1s, and (**f**) Si 2p, revealing a clear formation of metal and silicon oxides after phenol degradation.

Figure 9 shows the phenol removal and degradation rate constant of the Fe-based amorphous alloys with Fe-Cu couples fabricated from precursor with different Cu^2+^ concentrations. As shown in Figure 9a, the Fe_73.5_Si_13.5_B_9_Cu_1_Nb_3_ amorphous alloy with Fe-Cu galvanic couples exhibits excellent phenol degradation rate, which could complete decompose phenol in 3 min, with a kinetic constant (*k_obs_*) in the range from 0.94 to 2.35 min^−1^. Interestingly, it seems that the Fe-Cu galvanic couples will rapidly generate on their surface and significantly enhance the phenol degradation efficiency, as long as the Fe_73.5_Si_13.5_B_9_Cu_1_Nb_3_ amorphous alloy ribbons were dipped in the precursor. When the concentration of Cu^2+^ in the precursor increases from 6.25 mmol/L to 12.5 mmol/L, the kinetic constant will increase from 0.940 to 2.3396 min^−1^. Further increase in Cu^2+^ concentration will not show an obvious improvement, suggesting a moderate Cu^2+^ concentration will produce the most effective Fe-Cu couples for enhancing the catalytic activity of Fe-based alloys. In contrast to the extensive studies on Fenton and Fenton-like degradation of azo dyes, reports on phenol degradation using zero-valent metals including Fe-based amorphous alloys remain relatively limited. Existing literature shows that the reaction rate constants obtained with Fe-based amorphous catalysts typically fall within the range of 0.05–0.821 min^−1^, with the variations primarily attributed to differences in alloy composition and surface modification strategies, as shown in Table 2.

Wang et al. [38] further processed the Fe_78_Si_9_B_13_ amorphous alloy, which possesses intrinsically high catalytic activity, to fabricate microwires, thereby increasing the degradation rate constant for Rhodamine B to 3.7 min^−1^. However, the fabrication of such microwires is complex and costly, and the microwires are difficult to handle in practical applications. Zhang and Si et al. [29,30] applied surface modification treatments to the Fe_73.5_Si_13.5_B_9_Cu_1_Nb_3_ alloy and reported substantial improvement in azo dye degradation efficiency. Nevertheless, phenol exhibits physicochemical properties distinct from those of azo dyes, and these studies did not systematically evaluate the influence of environment factors on the degradation process. In the present work, the Fe_73.5_Si_13.5_B_9_Cu_1_Nb_3_ amorphous alloy decorated with micro/nano Fe-Cu galvanic couples demonstrates a markedly enhanced phenol degradation performance. The reaction rate constant increased from 0.16 min^−1^ to 2.36 min^−1^, representing a significant improvement achieved through a simple and scalable surface fabrication strategy. Moreover, the introduction of Fe-Cu galvanic couples also substantially enhances the catalytic activity of pure iron, highlighting the broad applicability and strong potential of this approach for practical wastewater treatment applications.

## 4. Discussion

**Figure 10:**
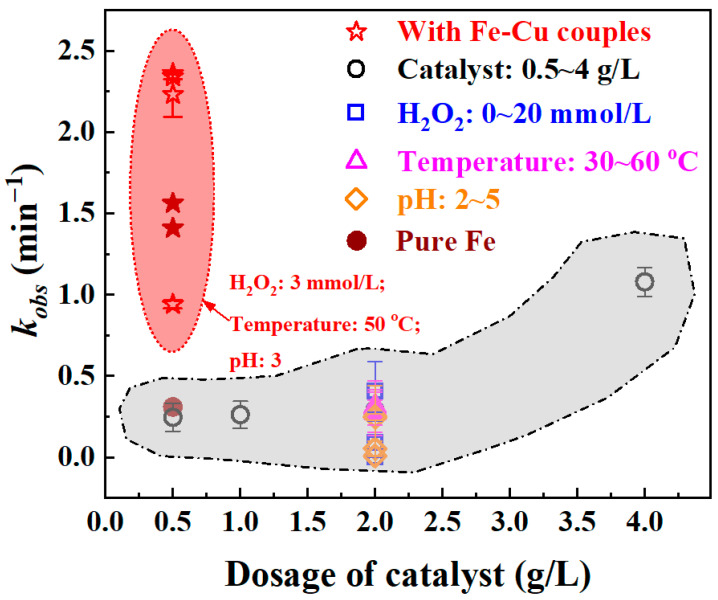
A comparison of kinetic constant *k_obs_* for phenol degradation between Fe_73.5_Si_13.5_B_9_Cu_1_Nb_3_ amorphous alloy with and without Fe-Cu couples (using hollow signs), combined with pure iron ribbons with and without Fe-Cu couples (using solid signs).

The Fe-based amorphous alloy exhibited remarkable catalytic activity for the degradation of phenol in coal chemical wastewater simulants. Figure 10 summarizes the degradation kinetic constants for the degradation of 50 mg/L phenol solution under various reaction conditions, including temperature, pH, H_2_O_2_ concentration, and Fe-based amorphous alloy dosage. The catalytic efficiency of phenol degradation was significantly influenced by the Fe-based amorphous alloy dosage. When the catalyst loading reached 4 g/L, the reaction rate constant (*k_obs_*) increased to 0.98 min^−1^, indicating decent degradation performance. In contrast, the Fe-based amorphous alloy modified via the in situ formation of Fe-Cu galvanic couples on its surface exhibited substantially enhanced catalytic activity even at a much lower dosage.

**Figure 11:**
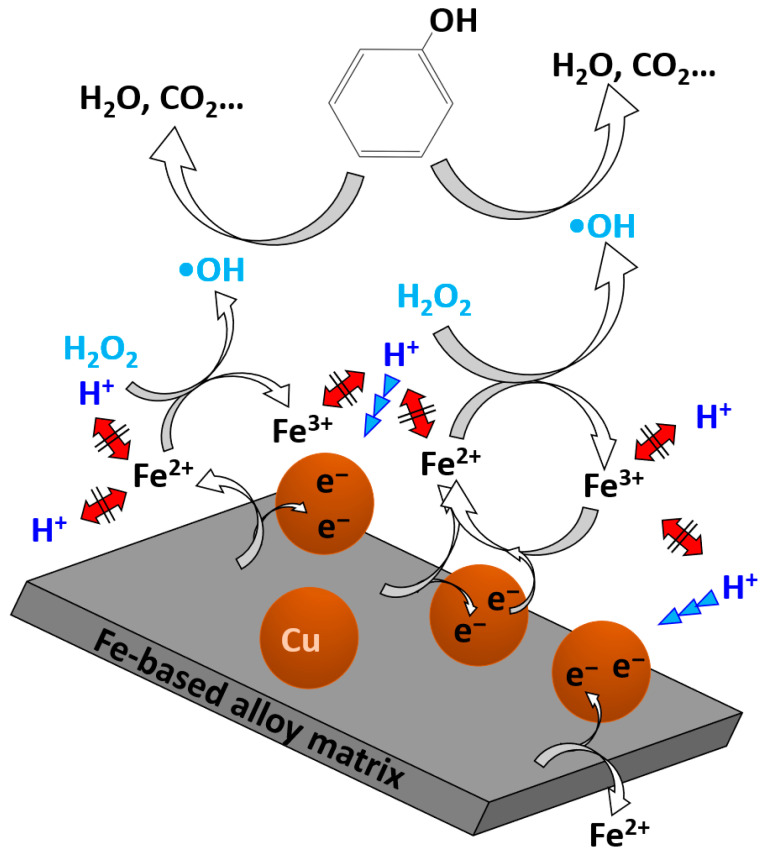
Schematic illustration of the plausible phenol degradation mechanism over the Fe-based alloy decorated with micro/nano Fe-Cu galvanic couples, revealing the electron transfer pathway and Fe^2+^/Fe^3+^ redox cycling. The red double-headed arrow with black lines indicates the electrostatic repulsion between Fe ions and H^+^, while the three blue triangles represent the attraction of electrons toward H^+^.

By precisely regulating the ionic concentration of the precursor solution during surface modification, the Fe-Cu-coupled amorphous alloy achieved exceptionally fast degradation kinetics, with reaction rate constants ranging from 0.94 to 2.36 min^−1^ at an alloy dosage as low as 0.5 g/L. Based on the systematic analysis of phenol degradation performance, surface morphology, and micro-structure evolution of the Fe-based alloys, a schematic illustration (Figure 11) was developed to elucidate the plausible degradation mechanism mediated by Fe-based alloys with micro/nano Fe-Cu couples. This pronounced enhancement can be primarily attributed to the formation of these micro/nano Fe-Cu galvanic couples, which facilitate rapid electron transfer between Fe and Cu and thereby accelerate the Fenton-like Fe^0^/Fe^3+^ redox cycle through efficient generation of hydroxyl radicals (·OH). In this system, ZVI (Fe^0^) donates electrons to adjacent Cu, leading to its dissolution as Fe^2+^. Due to electrostatic interactions, Fe^2+^/Fe^3+^ species accumulate around the Cu sites, which suppresses direct electron transfer to H^+^ and instead facilitates a more efficient Fe^2+^/Fe^3+^ redox cycling using electrons supplied through the Cu particles. Consequently, the enhanced production of hydroxyl radicals significantly accelerates phenol degradation, demonstrating the strong catalytic potential of Fe-based amorphous alloys modified with micro/nano Fe-Cu galvanic structures.

Moreover, the Fe_73.5_Si_13.5_B_9_Cu_1_Nb_3_ amorphous alloy modified by micro/nano Fe-Cu galvanic couples displayed excellent environmental adaptability. Under relatively mild conditions (H_2_O_2_ concentration = 3 mmol/L, temperature = 50 °C, and pH = 3), its catalytic efficiency was approximately one order of magnitude higher than that of the unmodified alloy. These results further confirm that introducing micro/nano Fe-Cu galvanic couples on the surface of Fe-based amorphous alloys provides a cost-effective, rapid, and highly efficient strategy for enhancing phenol degradation. Such improvements underscore the potential of atomic-scale fabrication in optimizing the catalytic interfaces of Fe-based amorphous alloys. This design concept not only offers new insights into the mechanistic understanding of Fenton-like reactions but also opens promising avenues for developing next-generation catalysts for industrial wastewater treatment.

## 5. Conclusions

In this work, a novel atomic-scale fabrication strategy was proposed to introduce micro/nano Fe-Cu galvanic couples onto the surface of Fe_73.5_Si_13.5_B_9_Cu_1_Nb_3_ amorphous alloy, thereby significantly enhancing their catalytic performance toward phenol degradation. This method enables the rapid and uniform formation of micro/nano Fe-Cu couples within only 15 s, providing an efficient route for surface modification. As a result, the phenol removal efficiency of the Fe-based amorphous alloys was improved by more than one order of magnitude, achieving complete degradation of phenol within approximately 3 min.

For the unmodified Fe_73.5_Si_13.5_B_9_Cu_1_Nb_3_ amorphous alloy ribbons, a distinct hatching period was observed at low catalyst dosages (0.5 to 1 mg/L). This limitation was eliminated following the introduction of micro/nano Fe-Cu galvanic couples, further demonstrating the superior catalytic reactivity and efficiency of the modified samples. Additionally, a quantitative relationship between phenol concentration (1 to 50 mg/L) and the UV-vis absorbance obtained from 4-aminoantipyrine colorimetry was established as *Y* = 0.4904 *X* − 0.7387, providing a convenient and reliable analytical basis for phenol quantification. The reaction parameters influencing phenol degradation were systematically investigated, and the optimal operating conditions for the Fe_73.5_Si_13.5_B_9_Cu_1_Nb_3_ amorphous alloy were determined to be a temperature of 50 °C, pH = 3, catalyst dosage of 4 g/L, and H_2_O_2_ concentration of 10 mmol/L. These findings can serve as a meaningful reference for future studies on Fenton-like degradation system towards phenol. Overall, the proposed atomic-scale fabrication approach offers a cost-effective, environmentally benign, and highly efficient strategy for phenol-containing wastewater remediation, holding great promise for future industrial-scale wastewater treatment application.

## Figures and Tables

**Table 1:** EDS results of Fe_73.5_Si_13.5_B_9_Cu_1_Nb_3_ amorphous alloy before and after phenol degradation.

	Elements (at. %)	Fe	Si	B	Cu	Nb	O	C
Fe-Si-B-Cu-Nb	Before degradation	84.05	8.85	0	1.97	5.13	-	-
	After degradation	41.92	11.97	5.53	8.06	0.28	16.2	0.29

**Table 2:** A brief summary of selected literature on phenol degradation using Fe-based alloys with/without surface modifications.

Catalysts	Organic Conc. (mg/L)	Catalyst Dosage (g/L)	pH	*K_obs_* (min^−1^)	Ref.
Fe-mZVAl^bm^	20	3	2.5	0.04	[35]
Fe_73.5_Si_13.5_B_9_Cu_1_Nb_3_	1000	-	-	0.05287	[36]
Fe_78_Si_9_B_13_	1000	-	-	0.33173	[36]
Fe_78_Si_9_B_13_	50	0.5	3	0.643	[5]
Laser processed Fe_78_Si_9_B_13_	50	0.5	3	0.821	[5]
Fe and FeO_x_	20 (p-chlorophenol)	0.2	5.6	0.268	[37]
Fe_78_Si_9_B_13_ microwires	25 (Rhodamine B)	0.5	3	~3.7	[38]
Surface activated Fe_73.5_Si_13.5_B_9_Cu_1_Nb_3_	20 (Orange II)	0.5	3	1.4–3.2	[29]
Fe_73.5_Si_13.5_B_9_Cu_1_Nb_3_ powder/Cu	20 (Orange II)	5	6.2	~0.0698	[30]
Pure Fe	50	0.5	3	0.30	This work
Fe_73.5_Si_13.5_B_9_Cu_1_Nb_3_	50	0.5	3	0.16–0.24	
Pure Fe with Fe-Cu couples	50	0.5	3	1.4–1.56	
Fe_73.5_Si_13.5_B_9_Cu_1_Nb_3_ with Fe-Cu couples	50	0.5	3	0.94–2.36	

## Data Availability

The original contributions presented in this study are included in the article. Further inquiries can be directed to the corresponding authors.
